# Extended Freeze-Dried BCG Instructed pDCs Induce Suppressive Tregs and Dampen EAE

**DOI:** 10.3389/fimmu.2018.02777

**Published:** 2018-11-29

**Authors:** Carla Lippens, Laure Garnier, Pierre-Marie Guyonvarc'h, Marie-Laure Santiago-Raber, Stéphanie Hugues

**Affiliations:** ^1^Department of Pathology and Immunology, School of Medicine, University of Geneva, Geneva, Switzerland; ^2^Tolerys SA, Buchillon, Switzerland

**Keywords:** experimental autoimmue encephalomyelitis model, plasmacytoid DCs (pDCs), regulatory T (Treg) cells, tolerance, BCG–Bacille Calmette-Guérin vaccine

## Abstract

Several clinical observations have shown that Bacillus Calmette-Guérin (BCG) vaccine has beneficial impact on patients suffering from different chronic inflammatory diseases. Here we evaluated whether BCG inactivated by Extended Freeze-Drying (EFD) which circumvents all the side effects linked to the live bacteria, could influence the development of experimental autoimmune encephalomyelitis (EAE), a mouse model for Multiple Sclerosis. EFD BCG strongly attenuates inflammation, both systemically and at the central nervous system (CNS) level, alleviating EAE. Mechanistically, EFD BCG directly impacts the phenotype of plasmacytoid dendritic cells (pDCs), and promotes their ability to induce suppressive IL-10 secreting regulatory T cells (Tregs) that inhibit encephalitogenic CD4^+^ T cells. When co-cultured with human allogenic naive CD4^+^ T cells, EFD BCG exposed human pDCs similarly induce the differentiation of IL-10 producing Tregs. Our study provides evidence that EFD BCG could be used as an immunomodulator of encephalitogenic T cells in multiple sclerosis patients.

## Introduction

Bacillus Calmette-Guérin (BCG) vaccine is prepared from an attenuated live strain of Mycobacterium bovis and has been used as a prophylactic vaccine against tuberculosis since 1921 (1). Despite its use as vaccine, BCG also displays anti-inflammatory properties in vaccinated people. For instance, a Japanese epidemiological study first reported that prevalence of allergic asthma in children immunized with BCG vaccine was significantly decreased compared to unvaccinated children (2, 3). Clinical studies performed later on adult cohorts supported that BCG vaccination might improve moderate-to-severe asthma (4–6) and had transient beneficial effects in Type 1 diabetes (7) and Multiple sclerosis (MS) (8–10). These different trials in MS patients provided evidence that a single administration of BCG vaccine (1) reduced MRI lesion activity of patients with relapsing-remitting MS (RRMS) (9), and (2) correlated with long-term beneficial effect of BCG treatment on tissue lesions (8). Indeed, BCG administration decreased the risk of new enhancing lesions and of persistent T1-hypointense lesion development over a 24-month period. Another study also reported that a single administration of live BCG vaccine after an episode of clinical isolated syndrome (CIS) significantly lowered the risk of relapses and diminished the probability of these patients to develop MS (10). From these observations emerged the concept that BCG could be used to treat chronic inflammatory diseases and particularly patients with CIS and relapsing-remitting MS. These clinical trials demonstrate interesting benefits from live BCG-treated patients but the presence of viable bacilli prevents its repeated use due to unwanted serious side effects. The use of heat-killed preparations of BCG or *M. vaccae* have already been considered in the past to treat asthma (11, 12), before being stopped at clinical development stage because of severe local adverse effects, including Th1 responses, and development of type IV hypersensitivity reactions.

Extended Freeze-Drying (EFD) preparation of Bacillus Calmette-Guérin vaccine circumvents these problems. EFD BCG results from a totally different process of bacteria killing, allowing the preparation of inactivated intact whole-cell BCG vaccine, unlike other sterilization processes that denaturate biological mycobacteria components. In contrast to live attenuated BCG vaccine, EFD BCG does not induce a Th1 delayed type hypersensitivity to mycobacterial antigens even at 10 times the vaccinal dose (13). Furthermore, whereas BCG vaccine cannot be administrated to immunocompromized patients due to risk of mycobacteria systemic dissemination, this side effect is not expected with inactivated bacilli of EFD BCG.

Preclinical studies reported high EFD BCG potency in the prevention and the protection of chronic inflammatory diseases. In allergic asthma, EFD BCG has been shown to reduce airway responsiveness and regulate lung inflammation (13–15). In colitis, prophylactic, and therapeutic injection of EFD BCG resulted in a significant reduction of symptomatic scores, body weight loss and inflammation (16). Finally, in atherosclerosis, EFD BCG significantly reduces atherosclerotic lesions (17). In all of these studies, EFD BCG therapy was correlated to the induction and/or expansion of regulatory T cells (Tregs) and IL-10 release. Importantly, EFD BCG administration did not induce adverse events such as weight loss or body temperature (13, 17), carcinogenic effect in a model of colitis (16), or interference with vaccines, and especially with the preventive effect of the BCG vaccine against *M. tuberculosis* (unpublished data). Thus, EFD BCG results in a product with enhanced anti-inflammatory potency of BCG vaccine and improved safety profile, without being a vaccine anymore.

Here we investigated in the experimental autoimmune encephalomyelitis (EAE) mouse model whether EFD BCG could be considered a novel therapeutic strategy in MS. This model is characterized by the induction of pathogenic Th1 and Th17 cells, whereas regulatory T cells (Tregs) were shown to play a protective role (18–21). We observed that mice treated with EFD BCG developed attenuated EAE. Both the severity and the incidence of the disease were dampened, and correlated with decreased peripheral and CNS inflammation, including a significant reduction of encephalitogenic Th17 cells. In addition, a particular subset of Tregs expressing CD103 and ICOS was increased in lymph nodes (LNs) draining the injection site and in the spleen. LN Tregs from EAE mice treated with EFD BCG demonstrated an increased suppressive potential *in vitro* and *in vivo*. Impact on Treg cells was due to a direct effect of EFD BCG on plasmacytoid dendritic cells (pDCs), which exhibited an activated phenotype and up-regulated Semaphorin 4A, a molecule implicated in maintaining Treg stability and function (22).

Overall, our results describe a novel mechanism by which a protective effect of EFD BCG treatment on clinical score development is mediated and which explains how the pDC functions are modulated to induce Tregs that inhibit autoreactive T cells. Similarly, human pDCs exposed to EFD BCG promote IL-10 producing Tregs *in vitro*, pinpointing EFD BCG as a feasible approach for MS treatment.

## Results

### EFD-BCG treatment dampens EAE pathogenesis

**Figure 1:**
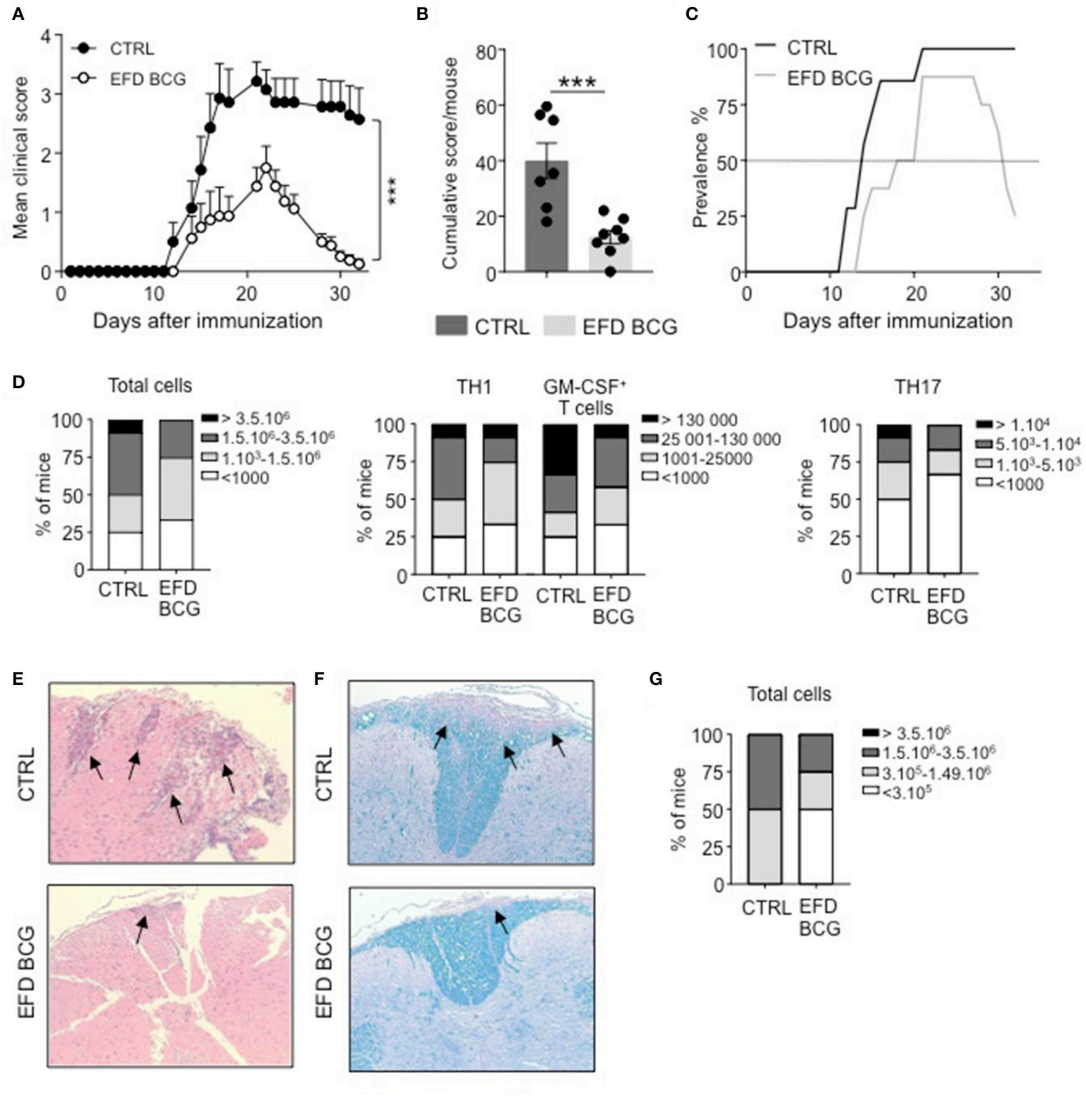
EAE is attenuated upon EFD BCG administration. **(A–G)** EAE was induced in WT control (CTRL) or EFD BCG treated mice. **(A)** Clinical scores were followed daily in untreated or EFD BCG treated mice. Cumulative scores **(B)** and EAE prevalence **(C)** were calculated for untreated control or EFD BCG treated mice during disease course. **(D)** Spinal cord (SC) infiltration at peak disease (D16 after immunization) in EAE control and EFD BCG treated mice. Frequencies of mice with rising number of total cells (left), CD4^+^ IFNγ^+^ T cells, and CD4^+^ GM-CSF^+^ T cells (middle), or CD4^+^ IL17^+^ T cells (right). Evaluations were performed based on total cell counting in SC extracts and flow cytometry frequencies of CD4^+^ T cells expressing either IFN-γ, GM-CSF or IL-17. Hematoxylin and eosin **(E)** and Luxol blue **(F)** staining of SC sections at peak disease (D16 after immunization) in EAE control and EFD BCG treated mice. **(G)** SC infiltration during early recovery phase (D21) after immunization in EAE control and EFD BCG treated mice. Frequencies of mice with rising number of total cells are depicted. Results are representative of at least 3 independent experiments with 7 mice/group **(A–C)** or data are pooled from three independent experiments with 7 mice/group **(D, G). (E, F)** Results show images representative of 4 mice/group. Statistical differences were determined via two-way ANOVA with Bonferroni *post hoc* test **(A)**. Error bars depict mean ± SEM. ^***^P < 0.001.

C57BL/6 females were injected subcutaneously with EFD BCG (100 μg) or PBS on the day of EAE induction by immunization with MOG_35−55_ peptide. The severity of EAE was significantly attenuated in EFD BCG treated mice, exhibiting clinical scores of 1.75 ± 0.37 (mean±SEM) at peak disease (day 16–20), compared to 3.21 ± 0.32 in controls (Figure 1A). In addition, the cumulative score, obtained by the sum of the daily EAE score per mouse, was significantly lower in EFD BCG treated animals (Figure 1B). Importantly, whereas disease prevalence was not affected at peak disease, it strongly decreased at later time point, with 75% of treated mice showing full recovery from EAE symptoms, compared to 0% in the control group (Figure 1C). Therefore, both disease severity at peak disease and prevalence during the recovery stage were improved following EFD BCG administration. Inflammation in the CNS was decreased at day 16 in EFD BCG treated mice, with reduced numbers of total CD45^+^ cells infiltrating the spinal cord (SC; Figure 1D), as well as decreased numbers of pathogenic CD4^+^ T cells producing IFN-γ, GM-CSF and IL-17 (Figure 1D). In line with reduced infiltrates in the spinal cord, HE and Luxol staining on SC sections 16 days after EAE induction demonstrated diminished numbers of inflammatory foci (Figure 1E) and demyelination (Figure 1F) in mice treated with EFD BCG. Pathogenic CD4^+^ T cells in the spinal cord were also decreased at later time point (day 21; Figure 1G), which is consistent with the noted improved recovery from EAE symptoms in EFD BCG injected mice.

**Figure 2:**
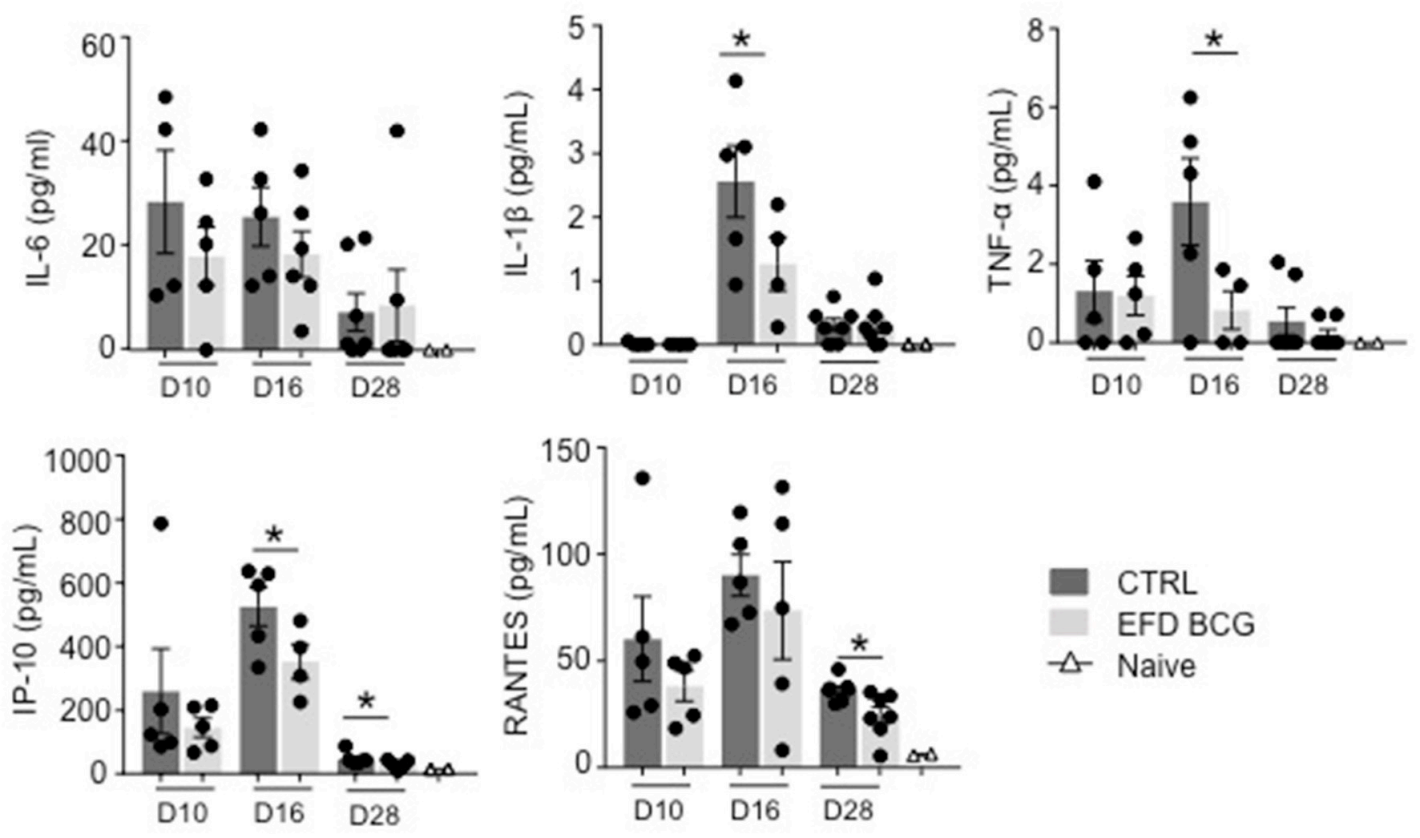
EFD BCG systemically restricts EAE-induced inflammation. EAE was induced in WT control (CTRL) or EFD BCG treated mice. Sera are sampled during priming phase (D10), at peak disease (D16), or in the later recovery phase (D28). Cytokines/chemokines were quantified by multiplex analysis in naïve mice, EAE control and EFD BCG treated mice. Graphs represent: upper panel IL-6 (left), IL-1β (middle), TNF-α (right); lower panel IP-10 (left), RANTES (middle). Results are representative of 2 independent experiments with 4–7 mice/group, and statistical differences were determined via a two-tailed Mann-Whitney test. Error bars depict mean ± SEM. ^*^P < 0.05.

EAE development results in the production of several circulating inflammatory mediators that can be measured in the blood, including IL-6, IP-10, RANTES and, to lower extent, IL-1β and TNF-α (Figure 2). EAE attenuation following EFD BCG administration correlated with reduced TNF-α, IL-1β, IP-10 serum levels at different time points after immunization, especially at day 16 (Figure 2). RANTES blood levels were also significantly reduced at day 28, whereas no difference was observed for IL-6 at any time points (Figure 2). Finally, systemic IL-10 levels were found undetectable in most of mice from both groups (not shown).

Altogether, our data show that EFD-BCG treatment dampens EAE disease course by modulating systemic inflammatory events induced upon disease development.

### EFD BCG treatment alters the treg/th17 ratio in secondary lymphoid organs of EAE mice

**Figure 3:**
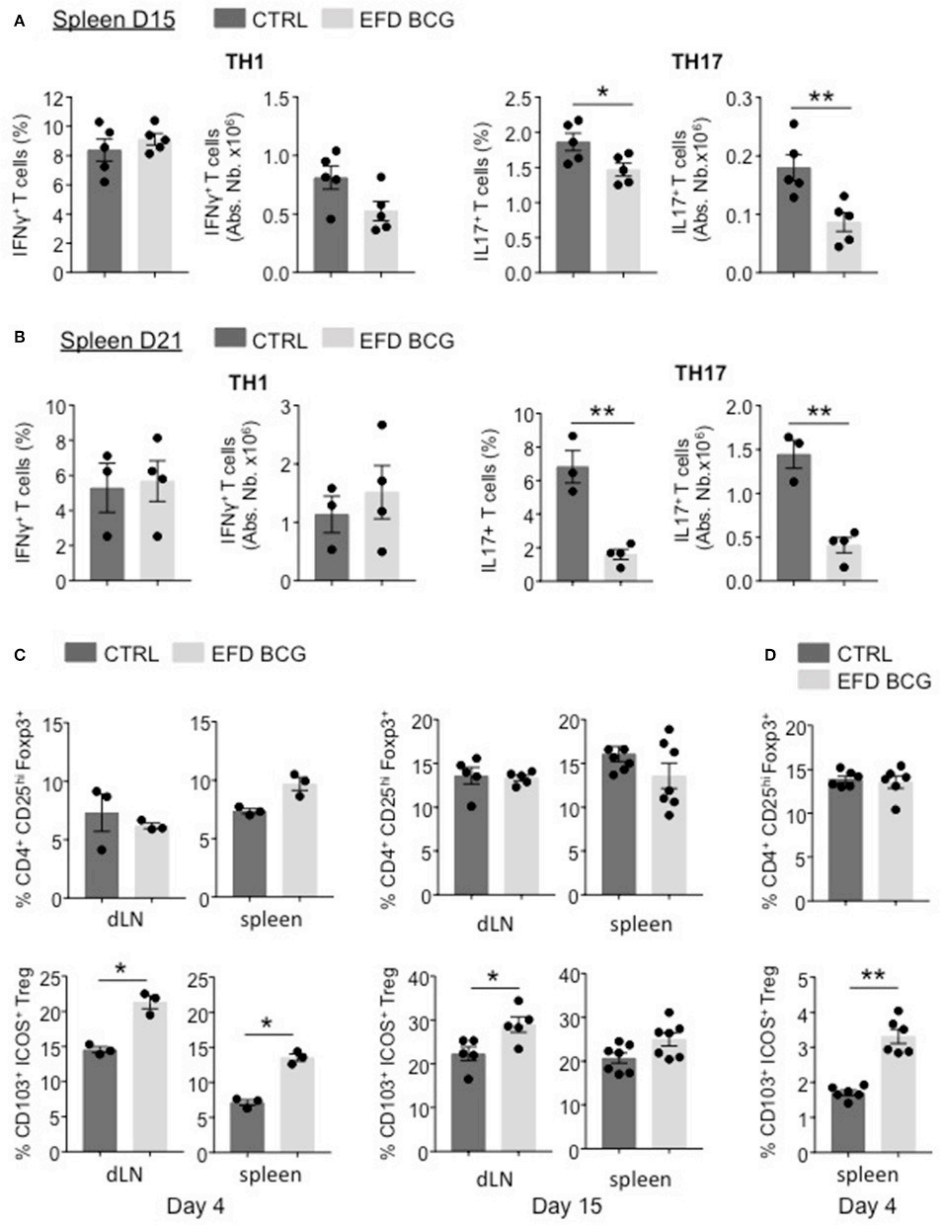
EFD BCG injection reduces encephalitogenic Th17 cells and promotes Tregs during EAE in secondary lymphoid organs. **(A–C)** EAE was induced in WT control (CTRL) or EFD BCG treated mice. **(A)** Frequencies and absolute numbers of IFN-γ^+^ (left) and IL-17^+^ (right) CD4^+^ T cells in spleen at peak disease (D15). Frequencies and absolute numbers of IFN-γ^+^ (left) and IL-17^+^ (right) CD4^+^ T cells in spleen during early recovery phase (D21). **(C)** Frequencies of total CD4^+^CD25^hi^Foxp3^+^ Tregs and of CD103^+^ICOS^+^ Tregs in draining lymph nodes and spleen during early priming phase (D4) (left) and at peak disease (D15) (right). **(D)** Frequencies of total CD4^+^CD25^hi^Foxp3^+^ Tregs and CD103^+^ICOS^+^ Tregs in spleens of WT mice 4 days after treatment EFD BCG. **(A–D)** Results are representative of two independent experiments with 3–7 mice/group. Statistical differences were determined via a two-tailed Mann-Whitney test. Error bars depict mean ± SEM. ^*^P < 0.05, ^**^P < 0.01.

EFD BCG administration led to a reduction of inflammation, which is not limited to the CNS but also takes place at the systemic level. Therefore, we characterized the pathogenic T cell responses in secondary lymphoid organs. At day 15 and day 21 post-EAE induction, whereas MOG_35−55_-specific Th1 responses were not affected in the spleen, both the frequency and the absolute numbers of splenic MOG_35−55_-specific Th17 responses were significantly impaired in EFD BCG-treated mice (Figures 3A,B). In parallel, although the Foxp3^+^CD25^hi^ Treg population was unchanged (Figure 3C), the frequency of Foxp3^+^CD25^hi^ Tregs expressing CD103 and ICOS, a subpopulation of Tregs being suppressive (20, 23), was significantly increased in LNs and spleen as early as day 4 after EAE induction in EFD BCG treated mice (Figure 3C). This phenotype was maintained at later time points (day 15 post-EAE), with significant augmentations of CD103^+^ ICOS^+^ Treg frequencies in LNs and a trend of increase in the spleen of EFD BCG treated EAE mice (Figure 3C). Since the frequency of total Tregs, as well as CD103^+^ ICOS^+^ Tregs, was in contrast not modified in the spinal cord (SC) by EFD BCG treatment (Supplemental Figure 1), we hypothesized that EFD BCG administration led to an expansion of CD103^+^ ICOS^+^ Tregs in secondary lymphoid organs that would inhibit encephalitogenic Th17 cells before they reach the CNS. Interestingly, increased frequencies of splenic CD103^+^ ICOS^+^ Tregs were also observed in non-immunized mice 4 days after EFD BCG injection (Figure 3D), conferring to EFD BCG a unique capacity to tolerize immune cells in a non-inflammatory environment.

### EFD-BCG treatment promotes suppressive tregs inhibiting EAE

**Figure 4:**
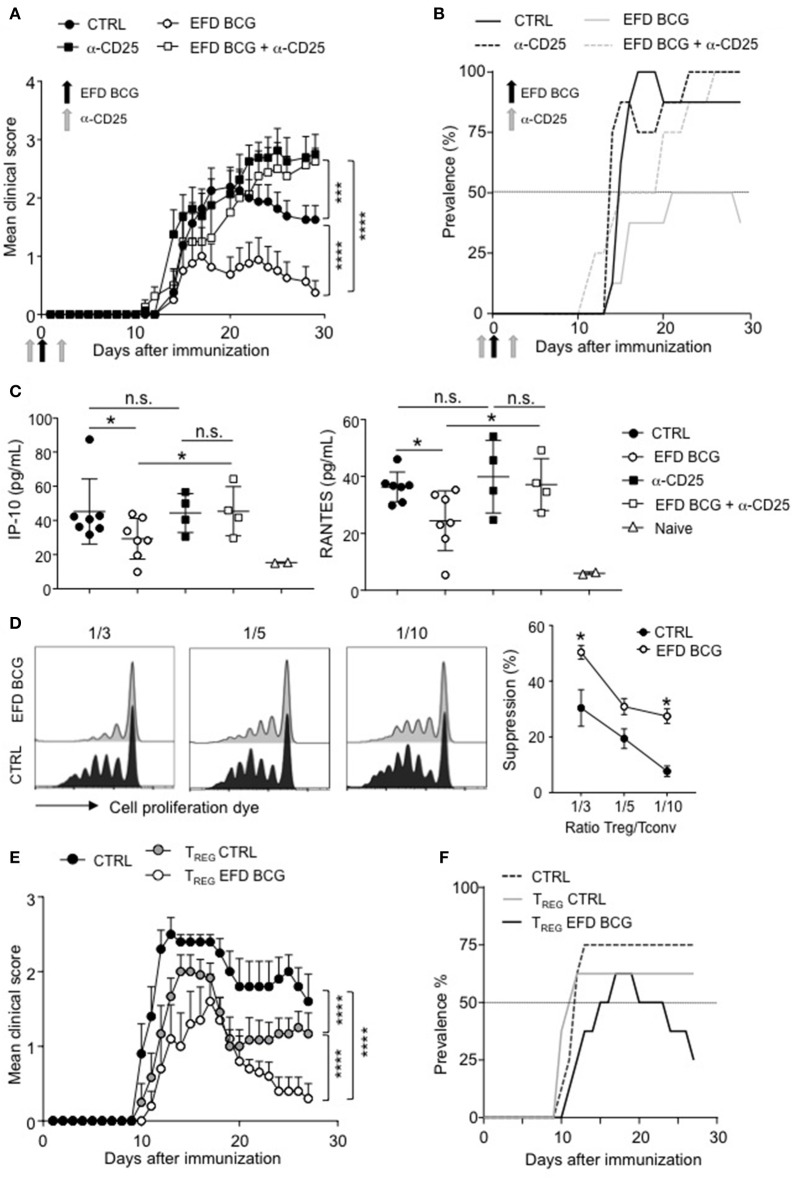
EFD BCG promotes suppressive Tregs that inhibit EAE. **(A–C)** Anti-CD25 antibody was injected or not in mice 4 days before and 2 days after EAE immunization. EAE was induced, and mice were treated or not with EFD BCG. **(A)** Clinical scores were followed daily in untreated control (CTRL), anti-CD25 treated, EFD BCG treated or EFD BCG + anti-CD25 treated mice. **(B)** EAE prevalence was calculated for untreated control, anti-CD25 treated, EFD BCG treated or EFD BCG + anti-CD25 treated mice during disease course. **(C)** IP-10 (left) and RANTES (right) concentrations were quantified by multiplex analysis in serum of untreated control, EFD BCG treated, anti-CD25 treated, EFD BCG + anti-CD25 treated or naïve mice in the later recovery phase (D28). **(D)** EAE was induced in WT control or EFD BCG treated mice. CD4^+^ CD25^hi^ cells were selectively sorted by flow cytometry and co-cultured with proliferation dye-labeled 2D2 CD4^+^ T cells and LPS activated MOG_35−55_ loaded cDCs. 2D2 T cell proliferation was assessed after 5 days. Histograms represent 2D2 T cell proliferation (left) and percentages of Treg-mediated suppression (right) for co-cultures with Tregs coming from EAE untreated control or EAE EFD BCG treated mice. **(E,F)** EAE was induced in RORγt-GFP x FOXP3-RFP mice and animals were treated or not with EFD BCG. After 10 days, CD4^+^ GFP^−^ RFP^+^ cells were purified from dLNs and transferred into WT recipients further immunized for EAE the day after. **(E)** Clinical scores were followed daily in EAE non-transferred control mice, EAE mice transferred with Tregs purified from untreated mice, and EAE mice transferred with Tregs purified from EFD BCG treated mice. **(F)** EAE prevalence was calculated during disease course for non-transferred control, EAE mice transferred with Tregs coming from untreated mice and EAE mice transferred with Tregs purified from EFD BCG treated mice. Results are representative of 2 independent experiments with 7 mice / group **(A, B, E, F)**, show data from 4–7 sera **(C)**, are representative of 2 independent experiments **(D)**. Statistical differences were determined via two-way ANOVA with Tukey's *post hoc* test **(A, E)**, one-way ANOVA with Bonferroni *post hoc* test **(C)** or two-tailed Mann-Whitney test **(D)**. Error bars depict mean ± SEM. ^*^P < 0.05, ^***^P < 0.001, ^****^P < 0.0001, n.s., non-significant.

We next assessed whether dampened EAE severity and improved recovery mediated by EFD BCG were dependent on Tregs. For that, we depleted the Treg population using anti-CD25 antibodies (intravenous injections, 4 days before and 2 days after EAE immunization). This treatment induced an efficient depletion of Foxp3^+^ CD4^+^ T cells in both control and EFD BCG injected mice (Supplemental Figure 2). Treg depletion in control EAE mice did not affect the incidence and the acute phase of the disease, but resulted in the abrogation of the recovery phase (Figures 4A,B), which is consistent with a role for Tregs in late time points of EAE (24, 25). EFD BCG protective effect on EAE mediated was abrogated by the depletion of Tregs, with both the incidence and severity reaching the levels of untreated mice (Figures 4A,B). In addition, Treg depletion reversed the inhibition of circulating IP-10 and RANTES levels (day 28) induced by EFD BCG (Figure 4C), indicating that Tregs induced following EFD BCG treatment are involved in the control of systemic inflammation induced during EAE.

To determine whether the phenotype observed in Tregs following EFD BCG treatment correlates with enhanced Treg suppressive functions *in vitro* and *in vivo*, we sorted CD4^+^CD25^hi^ cells by flow cytometry 10 days after EAE induction from draining LNs of mice treated or not with EFD BCG. *Ex vivo* Foxp3 staining confirmed that more than 90% of sorted CD4^+^CD25^hi^ cells belonged to the Treg population (Supplemental Figure 3). *In vitro* Treg suppressive assays demonstrated that Tregs isolated from EFD BCG treated EAE mice were more potent inhibitors of effector T cell proliferation compared to Tregs purified from control EAE mice (Figure 4D). As CD4^+^ T cells expressing CD25 do not exclusively belong to the Treg population, but to the effector non Treg T cells as well, we next used Foxp3-RFP^+^ x RORγt-GFP^+^ mice to perform *in vivo* adoptive transfer experiments (Supplemental Figure 4). Tregs (RFP^+^) isolated from PBS injected control mice conferred a significant inhibition of EAE, confirming already published observations (18, 20). In addition, Tregs from Foxp3-RFP^+^ x RORγt-GFP^+^ EAE mice were more protective, following transfer into EAE mice, when isolated from EFD BCG treated compared to control EAE mice. In particular, disease severity at peak disease and the prevalence of recovering mice were ameliorated (Figures 4E,F). Altogether, our results demonstrate that EFD BCG administration led to the generation of suppressive Tregs in secondary lymphoid organs, correlating with decreased peripheral inflammation and reduced generation of encephalitogenic T cells.

### EFD-BCG treatment promotes “pro-treg” plasmacytoid dendritic cells

Peripheral Treg induction during EAE has been linked to plasmacytoid dendritic cell (pDC) functions (18, 20, 26). Therefore, we next assessed the implication of pDCs in the protection mediated by EFD BCG in EAE.

**Figure 5:**
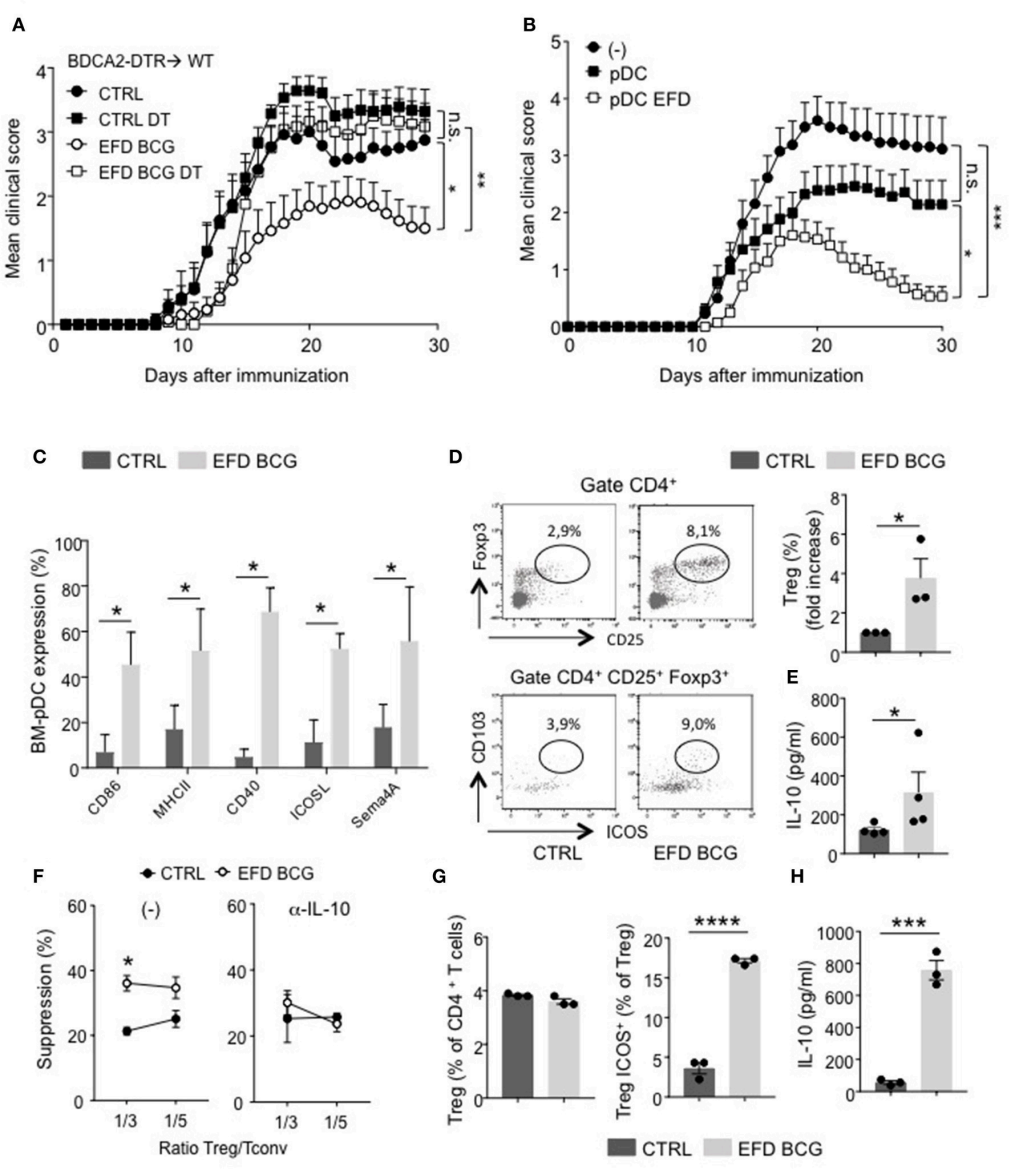
EFD BCG exposed pDCs dampen EAE and promote suppressive Tregs. **(A)** EAE was induced in BDCA2-DTR → WT chimeras treated (EFD BCG) or not (CTRL) with EFD BCG. Mice received or not 4 consecutives injections of DT every 3–4 days from D-1 to D10. Clinical scores were followed daily in untreated control, DT treated, EFD BCG treated or EFD BCG + DT treated mice. **(B)** EAE was induced in WT mice injected or not with untreated- or EFD BCG treated- BM-pDCs. Clinical scores were followed daily. Results are pooled from 2 independent experiments. **(C)** Purified BM-derived pDCs were treated *in vitro* for 16 h with EFD BCG. Expression levels of CD86, MHCII, CD40, ICOSL, and Semaphorin 4A were measured by flow-cytometry. **(D)** BM-pDCs, treated or not with EFD BCG for 16 h, were co-cultured with naïve CD4^+^ T cells for 72 h. Representative dot plots of Tregs analysis are shown. Treg enrichments are depicted as fold increase of Tregs frequencies between unstimulated vs. EFD BCG pre-activated pDCs-naïve T cells co-cultures. **(E)** IL-10 released from co-cultures were analyzed by ELISA and expressed in pg/ml. Data shown are pooled from three to four experiments performed. **(F)** EAE was induced in WT control or EFD BCG treated mice. CD4^+^ CD25^hi^ cells were selectively sorted by flow cytometry and co-cultured at indicated ratios with proliferation dye-labeled 2D2 CD4^+^ T cells and LPS activated MOG_35−55_ loaded cDCs in the absence (left) or in the presence (right) of anti-IL-10 antibody. 2D2 T cell proliferation was assessed after 5 days. Percentages of Treg-mediated suppression of T cell proliferation are indicated. **(G, H)** pDCs were purified from donor PBMCs, treated or not with EFD BCG for 16 h, and incubated for 6 days with naive CD4^+^ T cells isolated from a different donor. **(G)** Treg and ICOS^+^ Treg frequencies were evaluated by flow cytometry. **(H)** IL-10 production was measured in co-culture supernatant. Data are pooled from 2 experiments with 6–7 mice/group each **(A,B)**, are representative of 3 experiments **(C–E)**, are representative of 2 experiments **(F,G,H)**. Statistical differences were determined via two-way ANOVA with Bonferroni *post hoc* test **(A,B)**, two-tailed Mann-Whitney test **(C–F)**. Error bars depict mean ± SEM. ^*^P < 0.05, ^**^P < 0.01, ^***^P < 0.001, ^****^P < 0.0001, n.s., non-significant.

First we used BDCA2-DTR mice, in which pDCs can be significantly depleted following diphtheria Toxin (DT) injection (20, 27) (Supplemental Figures 5A,B). DT-treated mice were refractory to EAE attenuation mediated by the administration of EFD BCG (Figure 5A), suggesting that EFD BCG might modulate pDC phenotype and/or function. In order to test this hypothesis, we adoptively transferred bone marrow derived (BM)-pDCs treated or not with EFD BCG into mice in which EAE was induced the day after. As described previously (20), untreated pDCs moderately attenuated EAE compared to control EAE (Figure 5B). Moreover, EFD BCG pre-stimulated pDCs showed enhanced inhibition of EAE compared to untreated pDCs (Figure 5B).

Therefore, EFD BCG could affect pDC phenotype, both at steady-state and during EAE. *In vitro* treated EFD BCG BM-pDCs exhibited an activated phenotype, with a robust upregulation of MHCII, costimulatory molecules (CD80, CD40), regulatory molecules (ICOS), and, interestingly, Sema4A, which has been implicated in Treg stability and function (22) (Figure 5C). Apart from CD40 expression, which is upregulated by 40% of BM-cDCs exposed to EFD BCG, no significant difference in the expression of these molecules was observed between BM-cDCs exposed or not to EFD BCG (Supplemental Figure 6), with ICOS and Sema4A levels barely detectable, suggesting that the treatment preferentially modulates pDC phenotype. To determine whether a direct effect of EFD BCG on pDCs could be responsible for the impact on Tregs and the protection observed during EAE, BM-pDCs were incubated or not with EFD BCG, and co-cultured with naive CD4^+^ T cells. EFD BCG treated pDCs induced more Tregs and suppressive Tregs (ICOS^+^ CD103^+^) producing high levels of IL-10 than unstimulated BM-pDCs, conferring a tolerogenic phenotype to EFD BCG-stimulated BM-pDCs (Figures 5D,E). Finally, we assessed the implication of IL-10 in the enhanced ability of Tregs to supress effector T cell proliferation following EFD BCG treatment. We sorted CD4^+^CD25^hi^ cells by flow cytometry 10 days after EAE induction from draining LNs of mice treated or not with EFD BCG, and examined *ex vivo* their suppressive function in the presence or absence of an anti-IL-10 blocking antibody. We observed that Tregs isolated from EFD BCG treated EAE mice lost their enhanced T-cell inhibitory potential compared to Tregs purified from control EAE mice when IL-10 was inhibited during the co-culture (Figure 5F).

Altogether, these experiments demonstrated that EFD BCG modulates pDC phenotype toward a pro-tolerogenic phenotype, promoting IL-10 producing suppressive Tregs and resulting in EAE attenuation.

### EFD BCG treated human pDCs induce ICOS^+^ tregs and IL-10 production

To determine whether EFD BCG modulates pDC functions in human as well, pDCs were purified from peripheral blood mononuclear cells (PBMC) donors, treated or not with EFD BCG overnight, and co-cultured with naïve CD4^+^ T cells (ratio 1:3) isolated from distinct donor. After 6 days, the frequency of ICOS^+^ Foxp3^+^ cells was increased among total Tregs (Figure 5G), and IL-10 levels quantified in the culture supernatant were 10 times higher when naïve CD4^+^ T cells were co-cultured with EFD BCG-treated pDCs compared to those incubated with untreated pDCs (Figure 5H). Major fraction of IL-10 released from the co-culture assay come from Tregs, since a different pDC-naïve T cell ratio incubating 3 times less pDCs resulted in the same amount of IL-10 secreted (not shown). Therefore, similarly to mouse pDCs, EFD BCG impacts human pDCs by promoting their ability to induce suppressive Tregs *in vitro*.

### EFD BCG treatment during EAE acute phase induces transient disease remission

**Figure 6:**
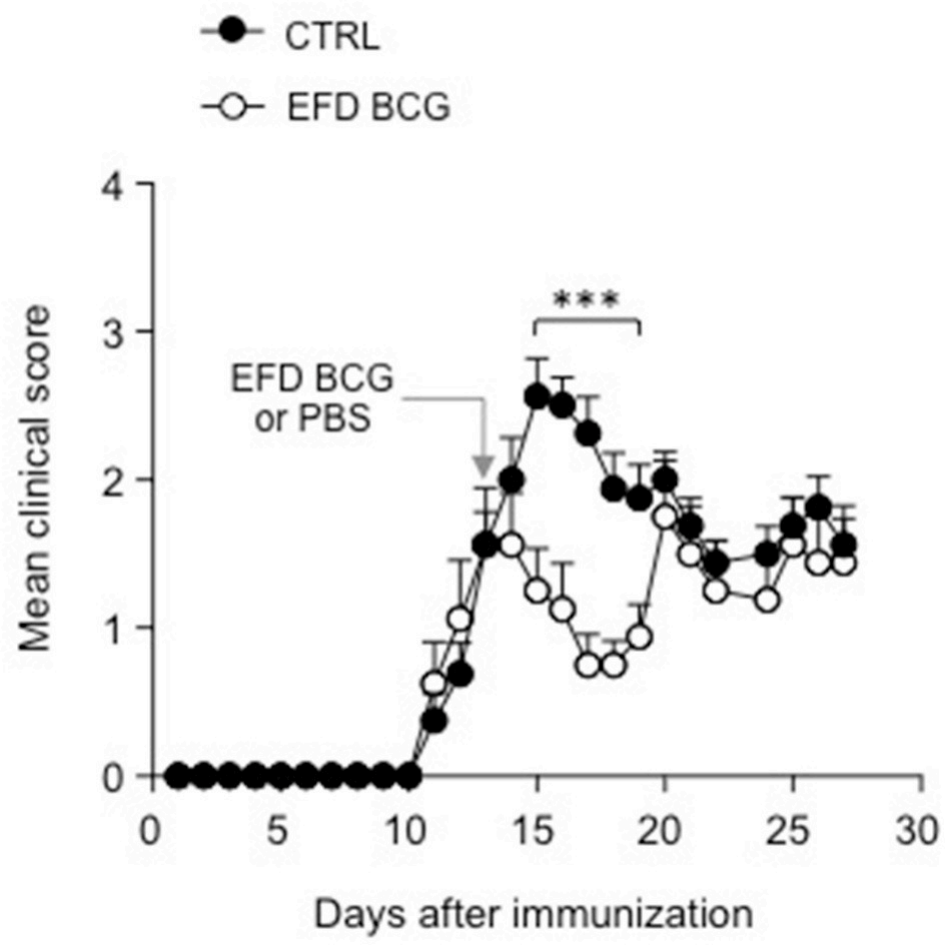
EFD BCG administration during disease acute phase transiently inhibits EAE. WT mice immunized for EAE were treated (EFD BCG) or not (CTRL) with EFD BCG at day 13. Clinical scores were followed daily. Results are representative of 2 independent experiments with 8 mice/group. Statistical differences were determined via two-way ANOVA with Bonferroni *post hoc* test. Error bars depict mean ± SEM. ^***^P < 0.001.

Finally, in order to explore the therapeutic potential of EFD BCG treatment in EAE, we treated mice after disease onset, 12 days after immunization with MOG_35−55_ in CFA. Whereas clinical scores of control mice continued to rise, those of EFD BCG treated mice rapidly decreased as soon as 2 days post-injection, resulting in a significant inhibition of EAE for several days (Figure 6). After a week of remission however, clinical scores of EFD BCG treated mice started to rise and reach those of control mice (Figure 6).

## Discussion

Here we show that EFD BCG administration alleviates EAE severity and promotes symptom recovery. EFD BCG treatment results in the dampening of systemic inflammatory events induced upon disease development, with reduced TNF-α, IL-1β, RANTES, and IP-10 serum levels. These results are consistent with previous observations in which serum levels of TNF-α and IL-1β were significantly reduced after EFD BCG treatment in various inflammatory mouse models such as colitis, allergic asthma and atherosclerosis (16, 17). In contrast to TNF-α and IL-1β diminutions at the peak of inflammation, serum levels of IL-6 showed some inconsistency from no changes to reduced quantities in EFD BCG-treated animals in the above-mentioned studies. Thus IL-6 does not represent a reliable marker for EFD BCG activity in contrast to TNF-α and IL-1β.

EFD preparation of BCG results in an inactivated intact whole-cell BCG vaccine, containing non-denatured biological mycobacteria components. EFD BCG avoids the side effects induced by live BCG or heat-killed preparations of BCG (13). Importantly, EFD BCG administration does not prevent further BCG vaccination. Indeed, EFD BCG-treated animals were able to develop normal Th1 and Th2 responses following infection with *Mycobacterium tuberculosis* and *Neisseria meningitides*, respectively (17). In addition, neither adverse event nor sensitization to purified BCG protein derivative was reported after EFD BCG injections (13, 17). Preclinical studies have already described EFD BCG as an anti-inflammatory product in various inflammatory contexts (13–15). In agreement, we observed the reduction of some cytokines in the blood of EFD BCG-treated mice, which is however not comparable to the decrease in EAE clinical scores observed. This suggests that additional effects might occur at the local level as well. Here we decipher the underlying mechanisms, by describing EFD BCG as an immunomodulator of pDC functions. EFD BCG-treated pDCs induce suppressive CD103^+^ ICOS^+^ Tregs that can inhibit encephalitogenic T cells. Interestingly, EFD BCG function as an immunomodulator in a non-inflammatory environment as well. Indeed, its administration to naïve mice similarly promotes CD103^+^ ICOS^+^ Tregs. Furthermore, steady-state mouse pDCs treated with EFD BCG inhibit EAE development, possibly, as we show *in vitro* and *ex vivo*, through the induction of IL-10 producing Tregs. Importantly, EFD BCG treated human pDCs also promote IL-10 producing ICOS^+^ Tregs. Therefore, similarly to mouse pDCs, EFD BCG impacts human pDCs by promoting their ability to induce suppressive Tregs *in vitro*. It has already been shown that human PBMC incubated with EFD BCG released augmented levels of IL-10 *in vitro* (28), although no functional assay was performed. Here we demonstrate a tolerizing effect of EFD BCG on human pDCs, which convert naïve allogenic T cells into ICOS^+^ effector Tregs.

Lastly, we could show that EFD BCG administration after disease onset is clearly capable of inhibiting established EAE, resulting in a transient remission phase. However, mice relapsed 7 days following treatment, suggesting that multiple EFD BCG injections might be required to induce long-term remission. Whether EFD BCG therapeutic effect involves mechanisms similar to those implicated when it is administered prior EAE induction remains to be determined.

Altogether, our study pave the way for further investigations on the use of EFD BCG, which, could be used as a preventive approach in patients diagnosed with a CIS episode, or, in addition to current therapies, could be envisaged to reduce the relapse rate in MS patients.

## Methods

### Mice

All mice had a pure C57BL/6 background and were bred and maintained under SPF conditions at Geneva medical school animal facility and under EOPS conditions at Charles River, France. BDCA2-DTR (29), RORγt-GFP x FOXP3-RFP (30), and 2D2 (31) mice have been previously described. WT C57BL/6N mice were purchased from Charles River (France). All procedures were approved by and performed in accordance with the guidelines of the Ethics Committee for Animal Experimentation of Geneva.

### Generation of BM chimeric mice

BDCA2-DTR chimeric mice were generated as described (32). Briefly, BM cells from BDCA2-DTR mice were recovered from tibia and femurs of donor mice. 5 to 7 × 10^6^ cells were injected intravenously into sub-lethally irradiated recipient mice (two consecutive doses of 500 cGy). Reconstitution was assessed by analyzing blood cells by flow cytometry after 6–8 weeks.

### EAE experiments

Active EAE was induced by immunizing mice subcutaneously in both flanks, with 100 μg of MOG_35−55_ peptide (MEVGWYRSPFSRVVHLYRNGK, Biotrend) emulsified in incomplete Freund's adjuvant (BD Diagnosis) supplemented with 500 μg/ml *Mycobacterium tuberculosis* H37Ra (BD Diagnosis). At the time of immunization and 48h later, mice also received 300 ng of pertussis toxin (Sigma-Aldrich) into the tail vein. Mice were monitored daily for disease clinical symptoms, and blindly scored as follows. 1, flaccid tail; 2, impaired righting reflex and hind limb weakness; 3, complete hind limb paralysis; 4, complete hind limb paralysis with partial fore limb paralysis; 5, moribund.

### Mouse treatments

EFD BCG is mechanically dispersed by steel beads and resuspended at 1 mg/ml in PBS. Mice are injected subcutaneously at the base of the tail with 100 μg of EFD BCG at the day of the immunization or when mean clinical scores reach 1.5. BDCA2-DTR mice were treated *i.p*. at indicated time points with 100 ng/mouse of DT. Anti-CD25 antibody (BioXcell) was administrated *i.v*. (100 μg/mouse) in mice 4 days before and 4 days after EAE immunization.

### *In vitro* BM derived DC generation

cDCs and pDCs were generated from BM of WT mice as previously described (18). Briefly, BM cells were recovered from tibia and femurs of mice and cultured, after red cell lysis, for 7–9 days in complete RPMI medium (10% heat-inactivated fetal bovine serum, 50 mm 2-βMercaptoethanol, 100 mm sodium Pyruvate and 100 μm of Penicillin/Streptomycin) supplemented with 100 ng/ml of murine Flt3L (PeproTech) for pDCs and with 20 ng/ml of GM-CSF for cDCs. For cDC culture, GM-SCF supplemented medium is added every 3 days and cells are split after 6 days of culture.

### Cell adoptive transfers

Adoptive transfers of Treg cells were performed as follows. RORγt-GFP x FOXP3-RFP mice were immunized for EAE as described above and injected or not with EFD BCG at the day of the immunization. CD4^+^ T cells were enriched from inguinal and axillary dLN cells (day 10 after EAE immunization) using a CD4^+^ T cell isolation kit (Miltenyi biotec). CD4^+^ GFP^−^ RFP^+^ Treg cells were isolated using a MoFlowAstrios (Beckman Coulter) and 1 × 10^5^ cells were injected intravenously into tail vein of WT recipient mice. EAE was induced by active immunization the day after Treg transfer.

Adoptive transfers of pDCs were performed as follows. 1.2–1.5 × 10^6^ BM pDCs derived from WT mice and stimulated or not with EFD BCG (20 μg) were loaded with 10 μg/ml of MOG_35−55_ and injected intravenously into WT recipient mice. EAE was induced by active immunization the day after.

### *Ex vivo* cell isolation

Treg cells were isolated from total skin LNs of naïve mice or from inguinal and axillary dLNs of EAE mice (day 10 after immunization). LNs were scratched and LN cells were subjected to CD4^+^ T cell enrichment using a CD4^+^ T cell isolation kit (Miltenyi biotec). CD4^+^ CD25^hi^ Treg cells were next sorted using a MoFlowAstrios (Beckman Coulter) or a Biorad S3 (Biorad).

### Co-cultures

WT BM-derived pDCs were generated *in vitro* for 7–8 days and purified using the Plasmacytoid Dendritic Cell isolation kit II (Miltenyi Biotec) according to manufacturer's instructions. Cell purity was assessed by flow cytometry and exceeded 90%.

For *in vitro* co-culture assay, purified BM-pDCs (1 × 10^6^/ml) were stimulated with 20 μg/ml EFD BCG in complete RPMI medium for 16 h; thus co-cultured with purified naive CD4^+^ T cells (EasySep Mouse CD4 T cell Isolation Kit; STEMCELL technologies, with addition of a biotinylated anti-CD25 antibody to the depletion antibody cocktail in order to remove activated/effector T cells) at a ratio 1/3 (33'000 DC/100'000 T) for 72 h. Supernatants are removed and kept at −20° until use and cells harvested, washed and stained with fixable viability dye, CD4, CD25, CD103, ICOS, FOXP3 to determine total Tregs and suppressive Tregs by flow cytometry on Gallios apparatus. Quantification of IL-10 in the supernatant of co-cultures was assessed by ELISA kit according to the manufacturer's information (Biolegends).

### Flow cytometry

Monoclonal antibodies used for flow cytometry were from: Biolegend; anti-CD11c (N418), Vβ11 (KT11), anti-IAb (AF6-120.1); from ThermoFisher: anti-CD4 (GK1.5 and RM4-5), anti-CD86 (GL1), anti-CD40 (1C10), anti-Foxp3 (FJK-16s), anti–IL-17 (ebio17B7), anti-IFN-γ (XMG1.2), anti-CD11b (M1/70), anti-CD11c (N418), anti-TCRβ (H57-597), anti-ICOS (C398.4A), anti-CD103 (2E7), anti CD16/32 (93), anti-CD25 (PC61.5), anti-ICOSL (HK5.3), anti-GM-CSF (MP1-22E9), Fixable Viability dye eFluor780, CellTrace Violet cell proliferation; from BD: anti-CD25 (PC61), anti-CD3 (145-2C11), and anti–IFN-γ (XMG1.2), anti-Sema4a (5E3/SEMA4A). For flow cytometry analysis, single cell suspensions were incubated with FcBlock (anti-CD16/32 FcγRII-RIII) for 10 min, at 4°C and stained with antibodies. Intracellular cytokine stainings were done using the Intracellular Fixation & Permeabilisation buffer set (eBioscience). For IFN-γ, IL-17, and GM-CSF staining, cells were first re-stimulated in complete RPMI containing PMA/ionomycin, and incubated 4 h at 37°C, 5% CO2. Golgi stop solution (BD Biosciences) was added to the last 2.5 h of culture. Data were acquired with an Attune NxT (Life Sciences) or a Gallios (Beckman Coulter) and analyzed using FlowJo software (FlowJo company).

### Multiplex analysis

Quantification of cytokines and chemokines was done on sera samples using a PPX-12 Mouse ProcartaPlex™ (Thermo Fisher) following supplier instructions. Briefly, standards and sera samples were plated with magnetic beads and incubated overnight at 4°C after a step for 30 min at RT, 500 rpm. Detection antibody mixture was added in each well, the following day, after a 30 min plate shaking and washing, and incubation was done for 30 min at RT, 500 rpm. Plate was washed and streptavidin-PE solution was added in each well and incubated for 30 min, RT at 500 rpm. Plate was washed again, felt with 120 μl of reading buffer, shaked at 500 rpm for 30 min at RT and ran on MAG-PIX Luminex instrument.

### Treg suppressive assay

WT, BDCA2-DTR or RORγt-GFP x FOXP3-RFP mice were immunized for EAE as described above and injected or not with EFD BCG at the day of the immunization. CD4^+^ CD25^hi^ T cells (or CD4^+^ GFP^−^ RFP^+^ Treg cells) were isolated from inguinal and axillary dLNs of EAE mice (day 10 after immunization) or from inguinal and axillary LNs of naive mice. 17 000, 10 000, or 5 000 Treg cells were incubated with 50 000 proliferation dye-labeled 2D2 CD4^+^ T cells (Treg:2D2 ratio: 1/3, 1/5, 1/10) and 50 000 LPS activated MOG_35−55_ loaded BM derived cDCs for 5 days. In some experiments, anti-IL-10 antibody (JES5-2A5) was added (10 μg/ml) in the culture. 2D2 T cell proliferation was assessed by flow cytometry. The percentage of Treg-mediated suppression was related to 0% of proliferation (relates to 100 % of suppression) in absence of cognate MOG_35−55_ peptide.

### Human pDCs and co-cultures with naive T cells

PBMC from Buffy Coats were first isolated with Ficoll Paque and pDC purified with Diamond Plasmacytoid Dendritic Cell Isolation Kit II human according to the manufacturer (Miltenyi Biotec). For *in vitro* co-culture assay, purified pDCs (60 × 10^4^/200μl) were stimulated with 20μg/ml EFD BCG in complete RPMI medium for 16 h in presence of human IL-3 (10 ng/ml, PeproTech), extensively washed, and co-cultured with purified allogenic naïve CD4^+^ T cells (Naive CD4^+^ T Cell Isolation Kit II human, Miltenyi Biotec) at a 1/3 ratio (16'000 DC/50'000 T) for 6 days in triplicates. Supernatants are kept at −20° until use and cells harvested, washed and stained with fixable viability dye, CD4, CD25, ICOS, Foxp3 to assess total Tregs, and suppressive ICOS^+^ Tregs by flow cytometry. Quantification of human IL-10 in the supernatant of co-cultures was assessed by ELISA kit according to the manufacturer's information (Biolegends).

### Statistics

Statistical significance was assessed by two-tailed Mann-Whitney test or by one-way ANOVA with Bonferroni *post hoc* test. EAE incidence was analyzed using two-way ANOVA with Bonferroni *post hoc* test. All statistical analyses were done using Prism 5.0 software (GraphPad Software). ^*^ *P* < 0.05, ^**^ *P* < 0.01, ^***^ *P* < 0.001, ^****^ *P* < 0.0001, n.s. = Non significant.

## Author contributions

CL, LG, and M-LS-R performed experiments, analyzed and interpret the data, and designed the figures. P-MG, M-LS-R and SH conceived and designed the study, analyzed and interpreted the data. M-LS-R and SH wrote the manuscript. All the authors reviewed and approved the final manuscript.

### Conflict of interest statement

The authors declare that the research was conducted in the absence of any commercial or financial relationships that could be construed as a potential conflict of interest.
