# Supplementary material for: Extended Freeze-Dried BCG Instructed pDCs Induce Suppressive Tregs and Dampen EAE
Source: Front Immunol. 2018 Nov 29;9:2777. doi: 10.3389/fimmu.2018.02777 (PMC6281986; doi:10.3389/fimmu.2018.02777)
Supplement: Supplementary file 1 [file Data_Sheet_1.PDF]

Supplementary Figure 1

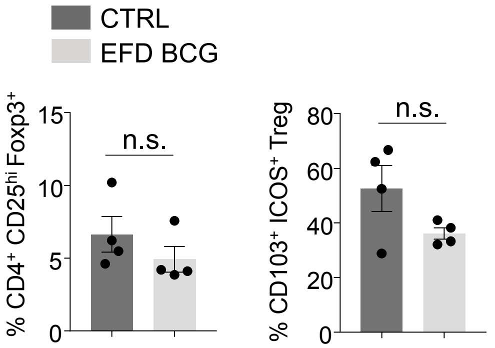

Supplementary Figure 2

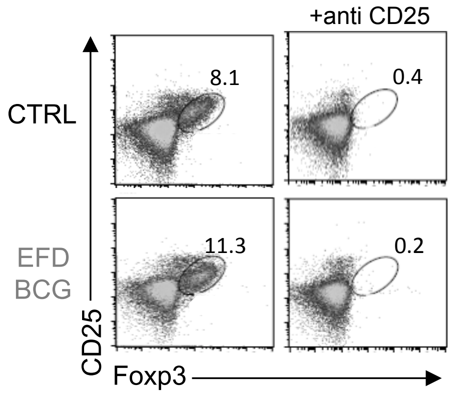

Supplementary Figure 3

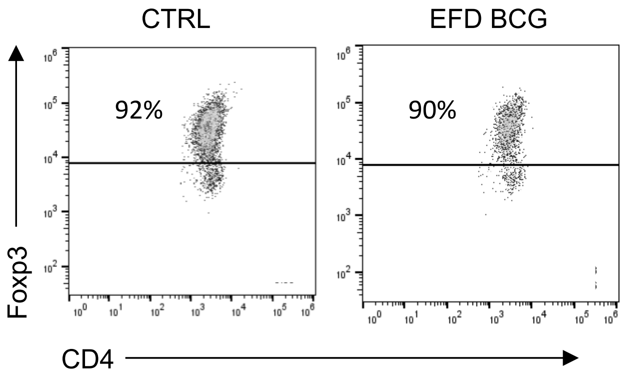

Supplementary Figure 4

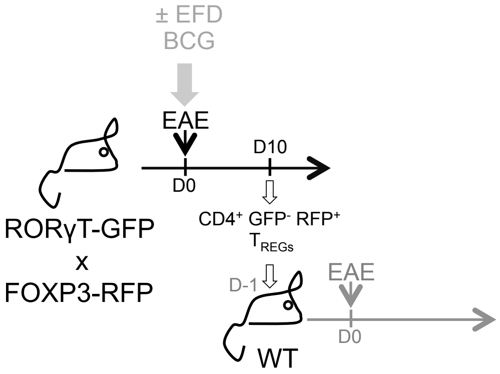

Supplementary Figure 5

A

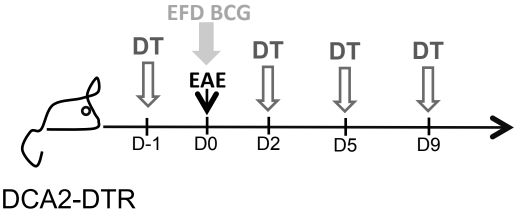

B pDC depletion (D11, dLNs) * → % of total cells

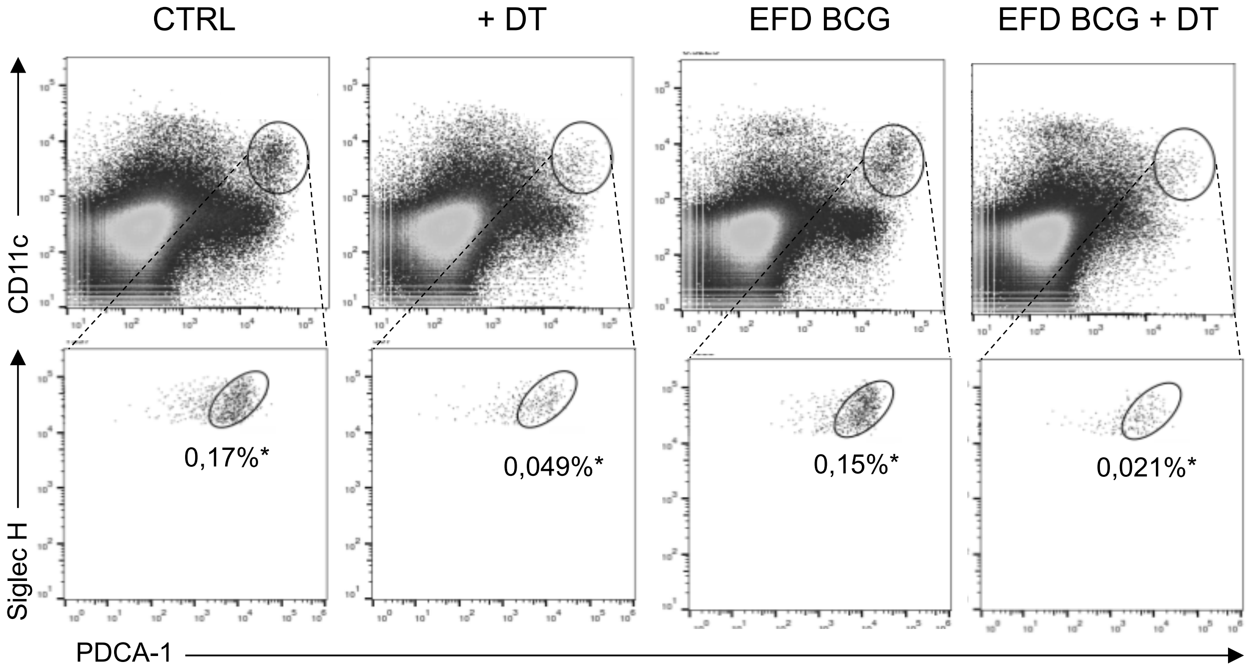

Supplementary Figure 6

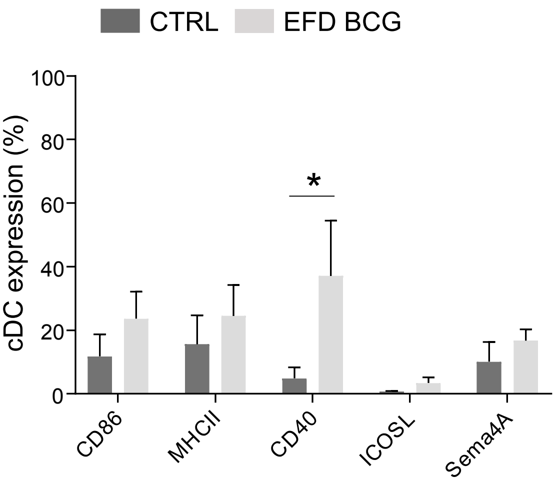

Supplemental Figure legends

Supplemental Figure 1**. Treg cells analysis in spinal cords**. EAE was induced in WT control or EFD BCG treated mice. At D29, Tregs from spinal cords were analysed by flow cytometry. Frequencies of % $CD4^+$ $CD25^{hi}$ $Foxp3^+$ among $CD4^+$ T cells and $CD103^+ICOS^+$ among $CD25^{hi}$ $Foxp3^+$ Tregs are indicated. Data are representative of three independent experiments with 4 mice/group. Statistical differences were determined by two-tailed Mann-Whitney test. Error bars depict mean ± SEM. n.s. $P > 0.05$.

Supplemental Figure 2. ***In vivo* Treg cell depletion in EAE mice**. Anti-CD25 antibody was injected or not in mice four days before and two days after EAE immunization and EFD BCG treatment. Frequency of $CD25^+Foxp3^+$ cells among $CD4^+$ T cells are depicted at D3 for EAE mice treated or not with EFD BCG and injected or not with anti-CD25 antibody.

Supplemental Figure 3. **Treg purification from LNs of untreated- or EFD BCG treated- EAE mice**. EAE was induced in WT control or EFD BCG treated mice. $CD4^+$ $CD25^{hi}$ cells were selectively sorted by flow cytometry at D10 and co-cultured with proliferation dye-labeled 2D2 $CD4^+$ $CD25^-$ T cells and LPS activated, MOG_{35-55} loaded, cDCs. 2D2 T cell proliferation was assessed after 5 days. Foxp3 expression in sorted cells from EAE control or EFD BCG treated mice.

Supplemental Figure 4. **Experimental design of Treg purification from RORgT-GFPxFoxp3-RFP EAE mice.** EAE was induced in RORγt-GFP x FOXP3-RFP mice and animals were treated or not with EFD BCG. After 10 days, $CD4^+$ GFP^- RFP^+ cells were purified from dLNs and transferred into WT recipients further immunized for EAE the day after. Experimental design is represented.

Supplemental Figure 5. ***In vivo* pDC depletion in EAE mice**. EAE was induced in BDCA2-DTR → WT chimeras treated or not with EFD BCG. Mice received or not 4 consecutive injections of DT every 3-4 days from D-1 to D10 **(A)** Experimental design is represented. **(B)** Frequency of $CD11c^+$ $PDCA1^+$ Siglec H^+ cells (pDCs) in dLNs at D11 for EAE mice treated or not with EFD BCG and injected or not with Diphteria toxin (DT).

Supplemental Figure 6. **EFD BCG in vitro treatment does not affect BM-cDC phenotype.** Purified BM-derived cDCs were treated *in vitro* for 16h with EFD BCG. Expression levels of CD86, MHCII, CD40, ICOSL and Semaphorin 4A were measured by flow-cytometry. Data are pooled representative of 3 experiments. Error bars depict mean ± SEM. $*P < 0.05$.
